# Chronopharmacology in Therapeutic Drug Monitoring—Dependencies between the Rhythmics of Pharmacokinetic Processes and Drug Concentration in Blood

**DOI:** 10.3390/pharmaceutics13111915

**Published:** 2021-11-12

**Authors:** Lukasz Dobrek

**Affiliations:** Department of Clinical Pharmacology, Wroclaw Medical University, 50-556 Wroclaw, Poland; lukasz.dobrek@umed.wroc.pl

**Keywords:** chronopharmacology, circadian rhythm, therapeutic drug monitoring, chronopharmacokinetics

## Abstract

The objective of the optimization of pharmacotherapy compliant with the basic rules of clinical pharmacology is its maximum individualization, ensuring paramount effectiveness and security of the patient’s therapy. Thus, multiple factors that are decisive in terms of uniqueness of treatment of the given patient must be taken into consideration, including, but not limited to, the patient’s age, sex, concomitant diseases, special physiological conditions (e.g., pregnancy, lactation, extreme age groups), polypharmacotherapy and polypragmasia (particularly related to increased risk of drug interactions), and patient’s phenotypic response to the administered drug with possible genotyping. Conducting therapy while monitoring the concentration of certain drugs in blood (Therapeutic Drug Monitoring; TDM procedure) is also one of the factors enabling treatment individualization. Furthermore, another material, and yet still a marginalized pharmacotherapeutic factor, is chronopharmacology, which indirectly determines the values of drug concentrations evaluated in the TDM procedure. This paper is a brief overview of chronopharmacology, especially chronopharmacokinetics, and its connection with the clinical interpretation of the meaning of the drug concentrations determined in the TDM procedure.

## 1. Introduction: Definition of Chronopharmacology and the Objective of the Review

Chronopharmacology is a field of science focusing on studying the effect of biological rhythms on pharmacotherapy, i.e., a branch of pharmacology studying the dependencies between the timing of drug administration and its effect [1,2,3]; it is one of the elements to be taken into consideration in broadly understood pharmacotherapy rationalization in order to ensure its maximum effectiveness and safety. Currently, therapy individualization in specific patients is based on consideration by the prescribing doctor of the age, sex, and individual physio- and pathophysiological conditions (existence of concomitant diseases) to anticipate the potential onset of drug interactions resulting from application of polypharmacotherapy—i.e., the patient’s pharmacogenetic profile (determined by way of evaluation of the phenotypic response to the administered drug or direct genotyping) [4]. The pharmaceutical care system and medicines use review (MUR service) provided by pharmacists also play a supplementary role in pharmacotherapy individualization [5]. Finally, the most optimal solution is implementation of personalized pharmacotherapy in the patient, taking into account the unique traits of the given patient and, additionally, specifying precisely the target for pharmacological intervention resulting from the analysis of the pathomechanism of the given condition [6,7]. The factors to be considered in pharmacotherapy individualization and personalization include also employment of monitoring of drug concentration in blood (Therapeutic Drug Monitoring; TDM) and consideration of the effect of biological rhythms on the pharmacokinetic disposition of the drug in the body and, consequently, on their pharmacodynamic properties [8]. Unfortunately, the issue of chronopharmacology remains a frequently overlooked and poorly studied aspect of therapy rationalization.

## 2. Aim of the Review

The objective of this short narrative overview is to provide a brief characterization of the chronopharmacological issues, with indications of the potential impact of biological rhythms on the rules of conducting therapeutical drug monitoring.

## 3. Chronopharmacology—A Brief Theoretical Overview

### 3.1. A Brief Historical Overview and Current Position in Pharmacology

Chronopharmacology uses the knowledge of biological rhythms to develop an optimal pharmacotherapeutic plan. A self-evident physiological feature of living organisms is the fact that biological phenomena are not invariable over time, but manifest rhythmicity—at the systemic, organ, and cellular level. Biological rhythms are self-supporting oscillations of physiological phenomena generated and controlled by endogenic “biological clocks”, characterized by repeatability. These rhythms are a manifestation of the adaptative capabilities of the body. They are activated to synchronize biological and behavioral functions with the dynamically changing and predictable conditions of the external environment, which affects the homeostatic status of the body [1,2,3,9].

The cyclic nature of the physiological phenomena studied by chronopharmacology was noticed already a long time ago. One of the first publications was the paper by a pharmacology professor at the University of Jena—Christoph Wilhelm Hufeland—who noted in 1797 that the basic rhythm determining the functioning of the body is the 24-h photoperiodism. Another important publication devoted to the biological role of circadian rhythms was the paper by a French researcher—Julien Joseph Virey—who observed in 1814 that “all medicines are not equally indicated effective given at different hours of the day” [10]. Further years brought studies on the cyclicality of heart rate (finally resulting in discovery of the phenomenon of Heart Rate Variability—HRV), changes in body temperature, respiratory rhythm, pain sensation, and exacerbation of symptoms of psychiatric disorders. Exploration of the issue of significance of biological rhythms in physiology and pharmacology continued over the years and was crowned in 2017 with the award of the Nobel Prize in Physiology and Medicine to three researchers—Jeffrey C. Hall, Michael Rosbash, and Michael W. Young—for their discovery of the basics of biological clock function in studies of fruit flies and their demonstration of the presence of proteins accumulating in cells at night and degrading during the day. In the official press release, the Nobel Assembly at Karolinska Institutet concluded that for many years, it has been known that living organisms, including humans, have an internal, biological clock that helps them anticipate and adapt to the regular rhythm of the day. The discoveries of Jeffrey C. Hall, Michael Rosbash, and Michael W. Young explained at a molecular level how the inner clock adapts our physiology to different phases of the day [11]. The research of Nobel laureates emphasized the significance of chronobiology and its fundamental importance in the development of chronopharmacology. However, the current meaning of chronopharmacology in pharmacological indications and rules of conducting TDM seem to be marginal. The above is also indirectly proven by the low count of scientific papers devoted to these issues. Review of the PubMed base carried out on 15 July 2021 using the search word “chronopharmacology” and without application of additional filters yielded only 399 records, including only 128 publications from the last 10 years. Most of those papers were published in the 1980s or 1990s. The combined search with the search words “chronopharmacology” or “chronopharmacokinetics” and “therapeutic drug monitoring” resulted in 12 records, including two articles from the last 10 years. Thus, the significance of biological rhythms and their relevance in clinical pharmacology must be emphasized to the greatest extent possible, including consideration of this phenomenon in the TDM guidelines.

### 3.2. The Features Characterizing Biological Rhythms: The Examples of Physiological Phenomena and Pathophysiological Conditions Characterized by a Chronobiological Background

**Figure 1:**
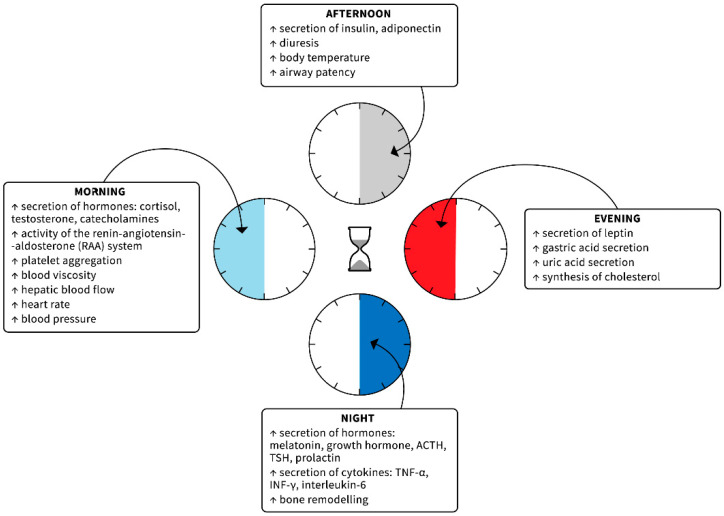
Physiological and pathophysiological phenomena that follow a circadian rhythm [1,2,3,9,12].

Biological rhythms are characterized by such features as period (duration of one full cycle), mean value (mesor), amplitude (difference between the mesor and maximum value), acrophase (time of reaching the maximum value during one cycle), and nadir (the opposite of the acrophase—time of reaching the minimum value by the rhythm). Taking the period into consideration, we can differentiate the ultradian rhythms, with cycles of varying duration, shorter than 24 h, ranging from one second through several seconds (e.g., oscillations in electroencephalographic recordings, heart rate, respiratory rate) to several hours (e.g., the basic sleep stage change cycle); circadian (“circa”—around; “dies”—day) rhythms, with an approximate duration of 24 h, related mainly to photoperiodism (e.g., sleep–wake rhythm, changes in core body temperature, secretion of selected hormones, changes in arterial blood pressure, and efficiency of the immune system); and infradian rhythms, the cycle of which exceeds 24 h (e.g., weekly, monthly, annual, and even seasonal) [1,2,3,9,12]. Physiological and pathophysiological phenomena controlled by the circadian rhythm are particularly noticeable. They are presented in Figure 1.

It must be also noted that the pathophysiology of many conditions, such as bronchial asthma, ulcer disease, rheumatic conditions, and depression is connected with disturbances of endogenic biological rhythms. Similarly, an increase in risk of onset of selected diseases at a specific time of the day is observed, for instance, cardiovascular episodes (sudden cardiac death, myocardial infarction, stroke) in the morning or exacerbation of peptic ulcer disease at night.

### 3.3. The Regulation of Biological Rhythms: The Genes That Control the Biological Clock

The circulatory system phenomena described above, along with multiple physiological functions, including the functions of other body systems, behavior, hormone levels, sleep, body temperature, and metabolism are regulated by the biological clock. The hierarchically superior central oscillator (“biological clock”) that coordinates the activity of other oscillators is the suprachiasmatic nucleus (SCN), located bilaterally in the anterior part of the hypothalamus, right above the optic chiasm. The activity of SCN is mostly modulated by sunshine. SCN receives the afferent information through the retinohypothalamic tract (originating from the photosensitive retinal ganglion cells) and from the other tracts: geniculo-hypothalamic tract, tracts leading from the structures of the reticular formation, septum, hippocampus, and limbic system. The impulsation reaches SCN that autonomically generates a cyclic activity modulated by the afferent signals. Efferent impulsation is transferred to the external oscillators—the target structures of the autonomic, endocrine, and immune systems that carry out secondary modulation of the functioning of other bodily systems, adjusting it to the rhythmic changes of the external environment—mostly the day–night rhythm. One of the most important tracts is the retino-hypothalamic tract connected with melatonin secretion from the pineal gland. The light inhibits secretion of melatonin, while the amount of this hormone increases at night. Melatonin receptors can be found in multiple peripheral tissues through which the hormone can exert its effect, modulating the physiological functions. Another important tract is the tract connecting the SCN with the periventricular nucleus of the hypothalamus, connecting the SCN with neurosecretory cells secreting corticoliberin (HPA tract—hypothalamus–pituitary gland–adrenal glands) and other cells controlling the endocrine glands [1,2,3,9,12].

According to the simplified description, at the molecular level, the cyclicity of the SCN physiological changes is induced by the oscillations in the expression of genes, their transcription factors, and the final synthesized proteins, which creates negative feedback loops with neuroendocrine output information. The main genes regulating the activity of the biological clock, being the stimulating fragment of the feedback loop, include *Clock* (Circadian Locomotor Output Cycles Kaput) and *Bmal1* (Brain-muscle Arnt Like-1). The said genes are transcribed and translated early during the day and the resulting CLOCK and BMAL1 proteins undergo heterodimerization and translocation to the cell nucleus, where they bind with specific DNA regions that are the promoter sections of genes *Per1*, *Per2*, and *Per3* (Period), and *Cry1* and *Cry2* (Cryptochrome). The target genes encode the proteins that are the negative effector limb of the regulation loop. During the next hours, PER and CRY proteins accumulate in the cytoplasm and, subsequently, are transported to the cell nucleus where they act as repressive transcription factors of the CLOCK–BMAL1 complex. At night, PER and CRY proteins are degraded, which stops their inhibiting effect on CLOCK–BMAL1 and, thus, initiates a new biochemical cycle [13,14]. As mentioned above, the discovery of the basics of circadian functioning of the biological clock and genes controlling it, connected with adaptation to the environmental conditions (amount of light) changing on a cyclic basis, was awarded the Nobel Prize in Physiology and Medicine in 2017 [11].

### 3.4. The Impact of Biological Rhythms on Pharmacology of Selected Diseases

The best-documented circadian rhythms include the circadian variability of arterial blood pressure. Both in normotensive persons and in most patients with primary arterial hypertension, decrease in the BP value and heart rate (HR) are observed at night, while their increase is observed in the morning hours, which is related to engaging in daily life activities. This rhythm is connected with the cyclic increase in the morning activity of the sympathetic nervous system, plasma renin activity, and secretion of hormones with a pressor effect, increasing the peripheral resistance and accelerating the automatism of the electrical conduction system of the heart in the morning. The blood pressure reaches its peak values in the late morning and early afternoon; after that, it declines between 8 p.m. and 2 a.m. when it is usually lowest [15,16]. Furthermore, fibrinolytic activity of the plasma is reduced in the morning, which is connected with increased tendency to formation of thrombi at that time. Thus, the morning period (3–4 h after waking up) is connected with an increased risk of cardiovascular events, such as acute coronary syndromes or strokes. An interesting observation was also the demonstration of the cyclic activity of the endothelium, with maximum secretion of nitrogen oxide in the morning and during the day, which is a physiological homeostatic mechanism counteracting the excessive increase in BP as a result of activity of the mechanisms referred to above. On the other hand, the evening and night are times of functional prevalence of the parasympathetic part of the autonomic nervous system, with decreased secretion of pressor hormones and activity of the RAA system decreasing the BP and HR values [17,18]. In clinical terms, the above phenomenon is expressed by the possibility of differentiation, based on the results of 24-h monitoring of BP changes in patients with primary arterial hypertension, of the “dippers” and “nondippers” subpopulation. In compliance with chronobiology observations, the expected decrease in the values of both systolic (SBP) and diastolic (DBP) arterial tension by 10–20% in relation to their values recorded during the day is typical for the “dippers”. Meanwhile, lack of the expected night SBP/DBP dip by at least 10% is a disturbance characteristic for the “nondippers”. On the other hand, a massive decline in SBP/DBP, exceeding 20% of their day values, is typical for the “extreme dippers” [18,19,20]. The phenomenon of “extreme dippers” is also connected with the risk of orthostatic hypotension as well as potential ischemic complications, including optic nerve damage [18]. Evaluation of the chronobiological morning increase in BP (“morning surge”) is also carried out in clinical conditions. Reference literature describes a positive correlation between the “morning surge” phenomenon and development of cardiovascular events as well as organ complications of the primary arterial hypertension. In practice, the value of the “morning surge” is established based on determination of the difference between the mean SBP within 2 h after waking up and the mean of three lowest night values of SBP. According to other recommendations, the “morning surge” is determined on the basis of evaluation of the mean BP from measurements taken 2 h after and 2 h prior to waking up. An excessive increase in the systolic pressure by ≥ 50 mm Hg and/or diastolic pressure by ≥ 22 mm Hg in the morning hours in relation to the mean pressure at night is considered pathological [21,22].

Primary hypertension is an excellent example of a disease that should be treated in line with chronopharmacotherapy rules to synchronize the changes in the hypotensive drug blood concentration with the 24-h change in arterial hypertension. This procedure enables improving the effectiveness and safety of antihypertensive treatment [23]. Thus, the general, routine recommendations for the chronotherapy of hypertension indicate that antihypertensive drugs should be administered in higher doses during the early-morning postawakening period, when BP is highest, and these agents should be delivered in lesser concentrations during the middle of a sleep, when BP is low. However, the detailed guidelines for the chronotherapy of hypertension depend on the precise characteristics of the hypertensive patient (“dipper”, “nondipper”, or “morning surge” patients) [15,16,17,18]. Several clinical studies have shown that administration of RAA-system-inhibiting drugs (angiotensin-converting enzyme inhibitors, angiotensin II AT1 receptor antagonists) at night—i.e., at the time of expected decreased activity—in nondipper patients translated to better hypertension control in comparison with administration of such drugs in the morning. Similar results have been obtained for thiazide diuretics applied in hypotensive monotherapy in the evenings. On the other hand, in the case of beta-adrenolytic drugs, it has been proven that administration of such drugs in the morning improves the efficiency of hypotensive treatment (due to the increase in catecholamines and expression of adrenergic receptors at this time of the day, as mentioned above) in comparison with evening dosage. It must be also noted that the dependency of change in hypertension therapy effectiveness depending on the timing of administration was not demonstrated in the case of dihydropyridine calcium channel blockers, most probably due to their rather long biological half-life [1,18,23,24].

The next adequate example illustrating the role of chronobiology is the use of melatonin and drugs modulating melatonin receptors in the treatment of insomnia. As mentioned above, melatonin is a hormone produced in the pineal gland under control of the circadian system in the hypothalamic suprachiasmatic nucleus (SCN). Normally, the melatonin level is low throughout the daytime, and rises in the evening as bedtime approaches. The plateau phase of melatonin secretion occurs during the night hours, and then declines by the typical wake time around dawn. With the evening melatonin rise, the circadian arousal level declines, reducing the homeostatic drive of daily activity. In this manner, the melatonin rise facilitates sleep onset and additionally reinforces the timing of the circadian system. It rationalizes the administration of melatonin as a chronobiological hypnotic drug [25,26]. Further, other melatonin receptor agonists (ramelteon, tasimelteon) are used to treat insomnia (late sleep induction) in selected European countries, Japan, and the USA [27,28]. In addition, agomelatine, used as an antidepressant (mentioned in Table 1 below), was evaluated for improving circadian rhythm and to induce evening sleep in patients with disturbed 24 h sleep–wake rhythms (e.g., in the course of dementia and depression) [29,30].

To sum up, according to the rules of modern pharmacotherapy, drugs used in many conditions should be, therefore, dosed optimally, taking into consideration the chronopharmacological criteria. The selected examples are presented in Table 1 [1,2,12,15,16,31,32,33,34].

To summarize, the basic physiological and behavioral functions of the body are subject to cyclic fluctuations as a result of the modeling impact of the central biological clock and peripheral oscillators. The modulation enables optimization of the homeostatic adaptation of the system to the dynamic changes of the environment. Comprehensive planning of pharmacotherapy should consider the impact of the said biological rhythms on the effect of drugs. In a broader sense, one can speak of chronomedicine as the task of not only adjusting the timing of drug administration to the circadian physio- and pathophysiological changes, but also implementing pharmacological intervention to restore normal biological rhythms (e.g., administration of melatonin as a hypnotic drug in jet-lag-type insomnia, phototherapy in seasonal depression, and others) [12]. Drug formulation technology includes also the design of therapeutic systems (enteric-coated systems, pulsatile cap systems, osmotic systems, diffucaps, time-controlled explosion systems, press-coated systems) that deliver the active substance over a predictable and expected time. Currently, the development of this type of technology regards, especially, hypotensive drugs [24,35].

## 4. Chronopharmacokinetics—Impact of Cyclicity of Biological Phenomena on the Pharmacokinetic Processes

As mentioned above, biological rhythms affect also the pharmacokinetic properties of drugs in the body. Thus, chronopharmacokinetics assesses the relationship between endogenous biological rhythms and the pharmacokinetic ADME processes. As the pharmacokinetic processes precede the drug finally reaching the target for pharmacological intervention, it must be assumed that chronopharmacokinetics (chronoPK) indirectly determines pharmacodynamic properties of drugs and, thus, drug concentration in blood determined in the TDM procedure. Therefore, taking the basics of pharmacokinetics into consideration allows even to distinguish chronoTDM [14,36].

The pharmacological effect of orally administered drugs is also indirectly determined by the circadian rhythm of the gastrointestinal tract that governs much of gastrointestinal physiology, including cell proliferation, motility, digestion, absorption and electrolyte balance or even fluctuations in intestinal barrier integrity, and composition and function of intestinal microbota [37]. Upon oral administration, the drug is absorbed depending on the physicochemical properties of the active substance (molecular mass, degree of ionization, degree of lipophilicity), physiological factors (visceral blood flow, gastric and intestinal pH, gastrointestinal motility, stomach emptying rate), as well as potential concomitant pathophysiological disorders. Gastric pH, which regulates drug ionization and solubility and indirectly determines the absorption via passive diffusion, presents circadian pattern, with the peak acid secretion just before midnight [38]. In the case of drugs for which the main transport mechanism through the biological membranes is not simple diffusion, bioavailability at the molecular level depends on the functioning of transport proteins found in the apical membranes of enterocytes, including such ATP-binding cassette (ABC)-type transport proteins as P-glycoprotein (P-gp), ABCB1, and MDR1 as well as breast cancer resistance protein (BCRP) and multidrug resistance-associated protein (MRP). The expression and transport functions of the said proteins are also subject to 24-h changes. Experiments have shown that the Bmal1 gene controls the cyclic expression of the MRP2 protein, through coordination of the secondary expression of DBP—an MRP2 activator, and E4BP4—a repressor protein. Regarding other transport proteins (e.g., BCRP and MDR1), it has been proven that their activity is also controlled by transcription factors (e.g., activating transcription factor-4; ATF4) subject to rhythmic changes [12,14]. In general, the absorption rate from the gastrointestinal system is higher during the day, especially in the morning and early afternoon, which depends on the increase in the visceral blood flow (secondary to the increased cardiac output and accelerated gastrointestinal motility at that time). This remark applies especially to lipophilic drugs—these agents are absorbed more rapidly after morning administration compared with evening administration, which leads to a higher “C max” and shorter “T max” during morning administration [12,39,40]. Gastric emptying after a meal and gastrointestinal motility demonstrate higher speed during the day compared with night, thus, also increasing absorption during the day [37]. In the case of NSAIDs, in quantitative terms, it has been proven that morning administration of diclofenac, indomethacin, and ketoprofen resulted in increase of the “C max” value by ca. 32%, 52%, and 50%, respectively [41]. A similar conclusion has been drawn in the studies evaluating the “C max” value of digoxin, nifedipine, propanolol, verapamil, terbutaline, and diazepam—a.m. administration of these drugs generated higher “C max” values compared with p.m. administration [42]. With regard to other drug administration routes, certain dependencies related to circadian changes have also been demonstrated, e.g., transcutaneous administration of melatonin in rats entailed higher bioavailability (both AUC value and “C max” were higher) if the drug was administered in the period of lower exposure of the animals to light. Similarly, subcutaneous administration of caffeine in animals generated higher bioavailability (an increased AUC and “C max”) in the case of administration in the early period of the night phase than in the case of administration after an hour of light phase [14].

The distribution stage is also subject to modulation by the rhythmically changing physiological phenomena. Distribution depends mainly on the blood flow, both in the central and peripheral compartments, and on the drug binding in the whole blood with the morphotic elements of blood (erythrocytes) as well as plasma proteins and tissues. The circadian changes in the cardiac output and visceral flows (hepatic, renal, muscular, and other) show that distribution of xenobiotics and drugs is the highest in the period of day activity. Binding of the blood proteins and tissues (albumins, alpha-1-acid glycoproteins, and other) is also subject to circadian fluctuations. Transcortin—a corticosteroid-binding protein—is characterized by the lowest binding capacity with endogenic and exogenic steroids at night and in the early morning (at ca. 4 a.m.), with the highest binding capacity at ca. 5 p.m., which may affect the relations between the free and bound fractions of these hormones [12,38,39]. Similarly, the plasma concentrations of albumins and alpha-1-acid glycoprotein are the highest in the afternoon and the lowest at night. It results from the aforementioned fact that an increase in the free fraction at night should be expected. However, the clinical significance of these chronopharmacokinetic circadian fluctuations regarding the capacity of protein to bind the drugs has not been described in detail yet [40].

Drug metabolism processes are subject to circadian fluctuations. The detoxification of drugs and other xenobiotics consists of three stages. During phase I, drugs are subject to biotransformation with the participation of oxidases, reductases, and hydroxylases. Phase II is aimed at conjugating drugs to a hydrophilic molecule in order to increase solubility and facilitate their excretion into urine, bile, and feces. These reactions are carried out mainly by sulfotransferases, UDP-glucuronotransferases, glutathione S-transferases, or *N*-acetyltransferases. Finally, in phase III, metabolites are transported outside the cell, into the body fluids (e.g., bile or urine) [38]. The most important physiological site of drug transformation is the liver, though it also takes place in extrahepatic tissues (kidneys, lungs, brain, and other). The hepatic clearance depends on the hepatic blood flow, intrinsic clearance (enzymatic activity of the liver), and the size of the free fraction of the drug (only the free fraction undergoes metabolic changes). As mentioned above, the hepatic blood flow changes according to the circadian rhythm; similarly, fluctuations in drug binding with proteins are observed [12,39,40]. In the case of drugs with a high hepatic extraction ratio (drugs characterized with high intrinsic clearance), the main factor determining the hepatic clearance value is the circadian variability in the hepatic blood flow (flow-dependent drugs). On the other hand, in the case of drugs characterized with a low intrinsic clearance value, the circadian variability of drug binding with proteins as well as activity of hepatic enzymes are the main factors determining the rate of their metabolism. The circadian fluctuations of concentration of such drugs in blood are particularly noticeable if administered in the form of an intravenous infusion [12]. The activity of hepatic cytochrome enzymes realizing phase I of drug biotransformation—CYP2B10, CYP2E1, CYP2A4, CYP2C22, CYP2E1, CYP4A3—changes over the day. Similarly, the activity of enzymes realizing the reactions of phase II (such as UDP-glucuronosyltransferase, glutathione S-transferase, *N*-acetyltransferase, epoxide hydrolases) changes according to the circadian rhythm. It has been described that oxidative transformations of phase I for hexobarbital, aniline, or imipramine are most accentuated during day activity and decrease at night, whereas the sulfation reactions are to the contrary—they reach their maximum activity at night [43,44,45]. At the molecular level, the circadian variability of drug metabolizing enzymes is related to the regulation of gene transcription that is intensified by the impact of the same xenobiotics. The transcription factors of the genes encoding hepatic enzymes are collectively referred to as xenobiotic receptors (e.g., constitutive androstane receptors (CAR), pregnane X receptor (PXR), PAS-domain helix-loop-helix transcription factor aryl hydrocarbon receptor (AhR)). CAR is highly expressed in the liver and small intestine, two key xenobiotic metabolising organs, and mediates the induction of expression of metabolising enzymes [46]. The said transcription factors are present in the cytoplasm of hepatocytes along with chaperones. Under the impact of xenobiotics (drugs), the compounds are translocated to the cell nucleus where they activate transcription of genes controlling the reactions of phases I and II [2]. Phenobarbital is a known CAR-related pathway inducer, increasing the expression of CAR target genes, while CAR activity is inhibited by androstanol and androstenol [46]. On the other hand, the cyclic expression of these genes is controlled by a set of transcription factors belonging to the PARbZip (PAR-domain basic leucine zipper transcription factors D-site-binding protein) family (hTEF—human transcriptional enhancer factor, and HLF—hepatic leukemia factor). Experiments have shown that mice deprived of PARbZip manifest lower expression of genes encoding phase I and II enzymes [47]. PARbZip factors are probably the most responsible for periodical binding to the promoter regions of the genes encoding enzymes involved in drug biotransformation and, indirectly, controlling CAR expression. Mice deprived of PARbZip manifested low activity of hepatic enzymes realizing drug biotransformation and lack of circadian differences in this activity [47,48,49]. It must be noted, however, that the post-translation processing of enzymes is also responsible for the final determination of their activity, as is confirmed by the results of some studies that prove time shifts in the enzymatic activity in the liver [14].

The process of excretion of the drugs and their metabolites is also characterized with circadian variability. Most drugs are excreted with urine, while some drugs are excreted with bile through the gastrointestinal system where they may additionally enter the hepatic portal cycle. The bile formation process involves mechanisms of secretion of bile salts and other organic anions (e.g., anion metabolites of drugs) by transporters of ABC class (ABCB11, ABCC2, and MRP2) and phosphatidylcholine (forming micelles with cholesterols and bile ducts) through MDR2 and ABCB4. Cation drug metabolites are excreted to the bile by means of MDR1 or ABCB1 [38,40]. The activity of these transport systems also undergoes circadian changes. It was demonstrated indirectly by the analyses of the circadian variability in the excretion of ampicillin in rats—aminopenicillin excreted predominantly through secretion to the bile [50]. Bile secretion has also been shown to be significantly marked in rats with experimental chronic bile drainage exposed to a regular light cycle (light from 6 a.m. to 6 p.m.), according to the preserved light–dark cycle [51]. The bile flow reaches higher values along with the increase in the concentration of lipids in the bile at the end of exposure of the animals to the 16-h light period and is lower in animals exposed to a 4-h light cycle [14]. The most water-soluble drug metabolites are eliminated through the kidney, and renal blood flow (RBF), glomerular filtration rate (GFR), tubular secretion and reabsorption, urine flow, and urine pH are the determinants of the process. The RBF is the main factor that accounts for GFR—about 20% of RBF is converted into urine through GFR [38]. Similar to bile flow, circadian fluctuations of these phenomena are demonstrated. GFR reaches the highest values during the day, declining at night; thus, in the case of drugs with a low degree of binding with proteins and for which glomerular filtration is the main factor determining the value of their renal clearance, the circadian fluctuations of GFR are the most important factor regulating the rate of their excretion [12,14]. The rhythmic oscillations of GFR are correlated with those of RBF—rhythmic RBF changes are probably entrained by the circadian arterial blood pressure and the cardiac output, with the peak during the active phase. However, circadian oscillation of GFR are not fully determined by only RBF changes, as GFR rhythm is maintained in bedridden patients or in transplanted kidneys; therefore, sympathetic drive is not implicitly required for this rhythm. These data suggest that GFR functional rhythmicity is also under control of intrinsic renal mechanisms, but they remain unknown [38]. Gentamicin administered in the early morning or in the early afternoon is characterized with lower nephrotoxicity (due to better drug elimination) in comparison with administration of this drug in the evening or at night [52]. Experimental studies demonstrated chronopharmacokinetic variations for valproic acid in mice. When administered *intraperitoneally (i.p.)*, this drug achieved higher plasma Cmax and AUC values in animals that received the dose in the active phase—dark period (after 7–9 h of darkness) and the parameters were found to achieve lowest values after administration in the rest phase—light period. The studies also demonstrated that the optimum tolerance to valproic acid in the tested animals, expressed by “survival rates”, occurred when the drug was administered in the second half of the light-rest span of mice, which is physiologically analogous to the second half of the night for human patients [53,54].

The urine pH value is also of some significance in drug excretion by the kidneys, having a secondary effect on the degree of ionization of the compounds dissolved in urine. In physiological terms, the urine pH value ranges from 4 to 8, which is controlled by the mechanisms of secretion and resorption of bicarbonate and hydrogen ions. The most important protein transporter involved in renal hydrogen ions secretion is the sodium-proton exchanger 3 (NHE3) located in the proximal tubules [38]. In the early morning hours, after the period of night rest, urine usually reaches lower pH values and, thus, has an indirect effect on ionization and increased resorption of acidic drugs from urine. Under low pH conditions, acid metabolites are not ionized, which facilitates their resorption from primary urine; this decreases their clearance value [38,40]. Other tubular transport phenomena (secretion/resorption), comprising the renal clearance mechanisms, are also characterized with circadian cyclicity, analogous to the transport mechanisms of the bile components mentioned above. The drugs and their polar, water-soluble metabolites are additionally transported to urine by means of ABP class transporters and the family of soluble SLC transporters located in the apical and basolateral membranes of the renal tubule epithelium [38]. Tubular transport in the nephron takes place mostly in the proximal tubules, with preferential transport of organic anions. Experimental tests have shown that mice deprived of PARbZip transcription factors manifest not only disturbances in hepatic cytochrome activity, but also reduced expression of tubular transporters (MRP4—ABCC4 and OAT2–SLC22A7) which, again, indicates the circadian background of variability in expression of these proteins [47].

To summarize, the circadian variability of drug effects may be observed, and the phenomenon is termed as “chronergy”; it results from circadian modulation of multiple biological functions, which affects the pharmacokinetic changes (chronoPK) and from the cyclically changing sensitivity and affinity of the targets for pharmacological intervention for drugs (which is termed as “chronesthesia”) [36]. Along with many other factors, chronoPK is, thus, an indirect essential element conditioning the effectiveness and safety of pharmacotherapy.

**Figure 2:**
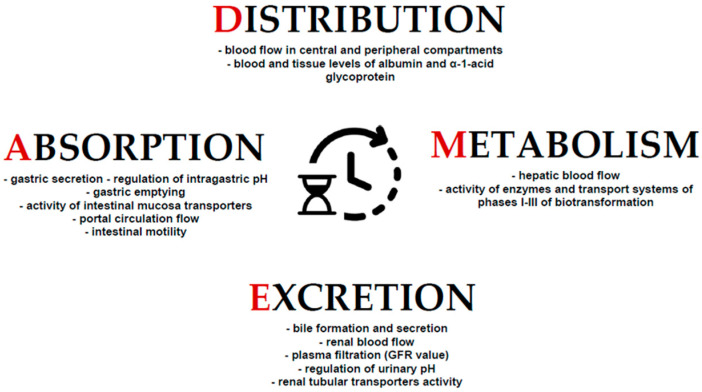
The summary of examples of chronobiological physiological phenomena that determine pharmacokinetic processes. The stages of ADME are controlled by phenomena characterized by the daily variability of their activity.

Figure 2 below presents a summary of the physiological processes showing diurnal variability and influencing the pharmacokinetic processes.

## 5. Therapeutic Drug Monitoring—Basic Assumptions and Rules

An essential method of individualization of treatment in the given patient, improving effectiveness and safety, is also the TDM method. TDM consists of determination of drug concentration (and, potentially, pharmacodynamically active metabolites of the drug) in the bodily fluids (usually in the whole blood, plasma, or serum; less frequently in saliva or, in the case of small children, capillary blood), along with clinical interpretation of the obtained result in the context of unique physiological and pathophysiological conditions of the given patient, which provides a valuable tool for dosage individualization. It must be noted that “measurement of drug concentration in blood” is not a synonym of “therapeutic drug monitoring” as mere analytical determination of drug concentration carried out in isolation from proper result interpretation is only a laboratory service, bringing no material information optimizing the pharmacotherapy (e.g., determined digoxin concentration must be considered in relation to the concentration of creatinine, calcium, and potassium; parameters of the acid–base balance; and presence of other drugs taken by the given patient) [55]. The primary TDM assumption is based on existence of dependencies between the drug concentration in blood and its effect (more precisely, the pharmacodynamic effect results from reaching a specific concentration within the area of the molecular site of drug effect). As measurement of drug quantity at the effect site (e.g., at the receptor level) is not possible, determination of its concentration in blood is a surrogate for such a determination. The TDM concept originates from the studies carried out in the 1960s that demonstrated for the first time the correlation between the plasma concentration of phenytoin and control of epileptic seizures [56] as well as the connection between the thymoleptic effect of lithium and its plasma concentration [57]. Subsequent years brought the development of TDM, especially in the scope of analytical techniques used to evaluate drug concentration. In the case of many, if not most drugs, TDM does not have to be implemented to secure their effect, as the pharmacodynamic effects may be easily evaluated clinically (e.g., measurement of temperature, BP, heart rate, glycaemia, lipid profile, 24-h urine volume, and other). On the other hand, in the case of drugs characterized with an end point that is difficult to assess (e.g., antiarrhythmic drugs having their own arrhythmogenic potential, antiepileptic drugs, behavior modulating drugs such as antidepressants, immunomodulating drugs), drugs manifesting nonlinear pharmacokinetics or significant individual variability of the pharmacokinetic profile as well as in the case of drugs with a low therapeutic index, TDM is a valuable tool, enabling the optimization of implemented treatment. Thus, TDM offers an option for evaluation of the safety of applied therapy (toxic effects of drugs, anticipation of potential onset of drug interactions), individualization of dosage ensuring the desired pharmacological effect, analysis of the patient’s “compliance” phenomenon, as well as explanation of the potential causes of pharmacotherapy failure (e.g., failure to take the drug, insufficient dosage, and others). In everyday clinical practice, TDM regards drugs that are commonly used in clinical practice, satisfying the criteria for implementation of monitoring of their concentrations in blood, as specified above. They are listed in Table 2 [31,55,58,59,60,61,62,63,64,65,66,67].

Table 2 presents the group of medications with the highest level of recommendation for TDM. In addition, it should be mentioned that in the case of many other drugs, especially in the field of neuro-psychopharmacology (e.g., aripiprazole, flupenthixol, quetiapine, duloxetine, fluoxetine, fluvoxamine, mirtazapine, paroxetine, sertraline, trazodone, venlafaxine), the TDM procedure also seems to be recommended [64,65,66,67]. Therefore, it can be concluded that the drug list for TDM has been progressively updated.

The sampling time is critical in the TDM process. According to the commonly adopted rules, the samples for analysis of the determined concentration of the most monitored drug must be collected at the elimination phase. Determination of drug concentration at earlier pharmacokinetic stages, with the absorption and distribution phases still in progress, is a source of unreliable results. In the case of drugs administered *per os*, blood for the analysis must be sampled no earlier than 2 h upon administration, where concurrent intake of food by the patient may extend the said time even more. In the case of slow drug distribution (e.g., upon oral administration of digoxin), sampling should take place 6 h after drug administration [60]. Thus, in practice, according to the adopted TDM recommendations, sampling must take place before the next dose is administered, and the drug concentration determined this way is referred to as “C through”. In certain clinical conditions, the peak (maximum) concentration (“C max”) is also assessed as clinically significant. C max measurements may be useful for some antibiotics, e.g., aminoglycosides or vancomycin. The antimicrobial effect of these antibiotics depends on the C max value in relation to the minimal inhibitory concentration. The general rationale for “C max” measurement is the treatment of patients with severe infections. C max is determined in these patients in order to verify the effectiveness of the antibacterial effect. Given the C max value, the parameter C max/MIC is calculated, which determines whether C max exceeds the MIC. Moreover, it is indicated for patients who are treated with other nephrotoxic and ototoxic drugs and in patients with impaired renal function. The time to take peak levels depends on the route of administration. The peak level is taken about 15 to 30 min after intravenous injections or infusions, 30 min to 1 h after intramuscular injections, and about 1 h after a drug is taken orally. Moreover, “C max” values are also determined for drugs used in high-dose treatment regimens in order to minimize the risk of developing dose-dependent serious adverse drug reactions. Routinely, blood samples must be collected from the treated patient for the purpose of determination of concentration of the given drug after determination of the stationary state, which usually takes place after a period equal to 4–5 biological half-lives of the evaluated drug. Reaching the stationary state may be accelerated with administration of the initial saturating dose. However, in the case of drugs characterized with long half-life, TDM can be implemented at any time if there is a risk of drug overdosing in the given patient (e.g., due to concomitant conditions of the metabolizing and eliminating organs such liver and kidneys). In the case of toxicity symptoms, the sample for the analysis should be collected and the determination should be carried out as soon as possible [55,56,57,58,59,60].

However, it should be emphasized that sampling time depends primarily on the purpose of monitoring of a particular drug and is also related to the evaluation of the safety and/or efficacy of the drug at any given time. It is worth remembering that the pharmacokinetics of the drug and the interpretation of the result obtained in TDM cannot be discussed in isolation from the pharmacodynamic aspects of the monitored drug and the overall clinical condition of the patient. In addition, the role of TDM and chronoPK in determining the values of pharmacokinetic parameters should be noted. The basic pharmacokinetic parameters include the following: clearance (a measure of the body’s ability to eliminate a drug); volume of distribution (a measure of the apparent space in the body available to a drug); half-life (the time required for the concentration of a drug to decrease by half after it has been distributed in the body); bioavailability (the amount of the drug reaching systemic circulation) [68]. Taking into account the fact that the values of pharmacokinetic parameters are calculated on the basis of the determined drug concentration, the daily fluctuations in drug levels may affect the values of the evaluated parameters. For example, the volume of distribution (V_d_) can be defined as the coefficient of proportionality between the measured blood concentration of the drug and the total amount of the drug in the body [69,70]. Since drug concentrations exert time-dependent fluctuations, the time of blood collection to determine the value of V_d_ and other parameters is of critical meaning. Thus, the TDM procedure allows taking into account the potential circadian variability of the basic pharmacokinetic parameters.

## 6. Chronopharmacology and Conducting a Therapeutical Drug Monitoring

According to the above theoretical outline of the TDM service, proper interpretation of the obtained result is critical in making specific therapeutic decisions. The interpretation must consider a number of individual physiological and pathophysiological conditions found in the given patient that could affect the final value of the determined drug concentration. Furthermore, as mentioned above, one of the most important factors is the time of collection of the sample for drug concentration analysis. According to the chronopharmacokinetic assumptions, drug concentration in blood changes following the circadian rhythm and, therefore, the determined drug concentrations in samples collected during the day and night may be expected to differ. Thus, the time of collection of the sample for the analysis may be of critical significance in the interpretation of the value of the determined concentration and the said fact must be taken into consideration in the TDM procedure protocol.

Studies concerning chronoPK of drugs routinely covered by TDM include both clinical and experimental reports. This chapter discusses the issues of chronoPK of selected examples of antiepileptic drugs, cardiovascular drugs, immunosuppressants, theophylline, and aminoglycosides.

In the case of antiepileptic drugs, in clinical studies, circadian changes in concentration of valproic acid (VPA) in urine were proven, with the maximum value noted between 2 and 6 a.m. and the lowest value recorded in the afternoon and evening. It was also connected with the lower rate of VPA metabolites (3-oxo-VPA and VPA glucuronides) elimination between 2 and 8 a.m. [71,72,73]. Daily fluctuations in the valproate concentration in plasma were assessed in 6 healthy volunteers who were given *per os* 300 mg of valproic acid sodium salt for 6 days at 9.00 a.m. and 6.00 p.m. Then, the basic pharmacokinetic parameters (first-order absorption rate constant—k_a_, absorption time t-lag, systemic clearance—CL, first-order elimination rate constant—K_e_, apparent volume of distribution—V_d_) were evaluated. In the case of the morning dose, a decrease in the absorption time t-lag was demonstrated. Moreover, k_a_ and the value of difference “C max”—“C min” were larger during the daytime compared with the nighttime. The systemic CL during the day did not differ from its value found at night. Therefore, the authors concluded that the timing of collection of the blood sample for determination of valproate concentration must be taken into consideration in valproate TDM in order to provide for possible fluctuations in drug concentration resulting from the chronopharmacokinetic aspects [74]. Chronopharmacokinetic variability was also proven for diazepam. The morning administration of this drug per os entailed reaching higher concentration in blood (as well as “C max” and “T max” values) compared with administration at night [71,75]. On the other hand, the experimental study showed that administration of carbamazepine in rodents ca. noon caused higher values of drug concentration (both “C max” and “C min”) in blood in comparison to morning administration [76]. Moreover, in the case of carbamazepine and valproate administered per os, it was shown in clinical assessment that chronopharmacokinetic changes were also diet-related. Having breakfast before the morning drug administration entailed reaching higher “C max” values and reduction of “T max” [77,78,79].

Another drug routinely covered by TDM is digoxin, for which circadian fluctuations affecting its concentration in blood were also proven. The clinical study carried out with participation of 10 patients suffering from congestive heart failure showed that the drug concentration reached the highest value 1 h after per os drug administration at 7.00 a.m. and that the said time was extended to 2 h when the drug was administered at 4.00 p.m. On the other hand, the average concentration of digoxin and AUC value were higher when the drug was administered at 4.00 p.m. in comparison with morning administration [80]. A similar 24-h dependency was demonstrated in another clinical study that evaluated the chronopharmacokinetic profile of digoxin in healthy volunteers administered 0.250 mg of the drug per os at 8.00 a.m. or 8.00 p.m. [81]; in this study, blood samples were collected at specific times for 48 h after each timed dose and “C max”, “T max”, the time to reach “C max”, area under plasma concentration curve AUC, and elimination half-time T1/2 of digoxin were determined. Similarly, morning drug administration resulted in the reduction of time “T max” with an accompanying upward trend in the C max value. Other parameters of digoxin pharmacokinetics demonstrated no administration time dependency [81]. Again, reaching higher concentration of digoxin in blood after morning administration per os of 0.125 mg of the drug was shown in the study by Kopecka et al. [82]. The researchers revealed significantly higher “C min” concentration directly prior to administration of the morning dosage as well as significantly higher “C max” after morning administration (increase in the range of drug concentration in blood). The other parameters: AUC, “T max”, and the total plasma clearance of digoxin did not differ. Therefore, they concluded that morning administration of the drug entails higher fluctuations of digoxin concentrations in comparison with evening administration. Chronopharmacokinetic changes were also analyzed for procainamide. One of the clinical studies evaluating the chronopharmacokinetic profile of the drug administered per os at 10.00 a.m. or 8.00 p.m. in the dose of 500 mg in patients with premature ventricular beats did not show any statistically significant differences in the scope of concentration of the drug or its active metabolite (*N*-acetylprocainamide) in blood as well as other assessed pharmacokinetic parameters (AUC, elimination half-life, or total clearance) [83]. However, one of the experimental studies showed that *i.p.* administration of 50 mg of procainamide in rats exposed to the 12-h light–dark cycle at 4.00 a.m., 10.00 a.m., 4 p.m., and 10 p.m. demonstrated a significant difference in the procainamide metabolism rate, affecting the drug concentration in blood. Highest fluctuations were noted when the drug was administered at 10.00 a.m. (highest elimination half-lives were found at that time) [84]. Another drug frequently covered by the TDM procedure is theophylline. Chronopharmacokinetic changes that could affect the evaluated drug concentration in blood were also shown for this drug. In one of the clinical studies, eight healthy volunteers were administered theophylline at a dose of 5 mg/kg of body mass per os at 9.00 a.m. and 9.00 p.m. The drug concentration in blood measured 0.5 h after its morning administration was significantly higher than in the case of the evening dose. Concurrently, no significant differences in pharmacokinetic parameters describing the elimination stage (total clearance, half-life) were demonstrated, which suggests that changes in drug concentration in blood are determined mainly by the chronobiology of the absorption process [85]. A similar trend in changes was shown in other studies. Patients treated with solid theophylline formulation were demonstrated to have morning “trough” theophylline concentrations at steady state 10–16% greater than corresponding evening “troughs” and the difference was statistically significant. However, the authors emphasized that this phenomenon varies on a case-by-case basis to a great extent. However, the final conclusion of the study was the statement that 24-h variability in theophylline concentration in blood requires blood sampling at the same time of the day when monitoring its concentration in blood [86]. The results of the above study were also confirmed in another clinical study evaluating the blood concentration of theophylline administered in the form of modified-release preparation in eight healthy volunteers at 8.00 a.m. or 8.00 p.m. for 4 days. The mean concentrations of theophylline in blood assessed 4 and 8 h after drug administration were 40% higher in the case of morning dosage [87]. The chronopharmacokinetic aspects were also assessed for immunosuppressive drugs. An experimental study evaluated the blood levels of mycophenolate mofetil in rats exposed to a 12-h light and 12-h dark cycle. The drug was administered in animals by *i.p*. route at the dose of 200 mg/kg body weight at either of the four different circadian stages (1, 7, 13, and 19 h after Light Onset; HALO). The highest and lowest values of Cmax were obtained when mycophenolate mofetil was injected at 7 and at 19 HALO, respectively. The highest and lowest mean values of plasma clearance were demonstrated at 19 and at 7 HALO, respectively. “Tmax” of mycophenolate mophetil remained similar regardless of the circadian time of injection of the drug. The mycophenolate mophetil concentration in blood of studied animals was, thus, characterized with circadian differences. The authors concluded that the mechanism of circadian rhythm in mycophenolate tolerance might be partly explained by the circadian variation of pharmacokinetics, since the time (7–13 h after light onset) of maximum hematological and digestive toxicity of the drug corresponds to that of the lowest plasma clearance and the highest “C max” and AUC0-24 of the drug [88]. Tacrolimus concentration in blood was assessed in transplantology patients (kidney recipients) treated per os twice daily (every 12 h). In this study, whole blood and intracellular tacrolimus concentrations over a period of 24 h (an intensive sampling at 0, 0.5, 1, 1.5, 2, 3, 4, 6, 8, 12, 12.5, 13, 13.5, 14, 15, 20, and 24 h) were carried out. Whole blood and intracellular AUC12–24 h and “C max” achieved after tacrolimus night dose was significantly lower than after morning dose administration [89]. The next clinical study that also included transplantology patients demonstrated a similar dependency for the evaluated cyclosporine concentration—the drug reached higher concentrations (“C min” and “C max”) in the patients’ blood when administered orally in the morning compared with evening administration. As suggested, the concentrations of both cyclosporine and tacrolimus must be determined 2 h after administration of the morning dose, whereas collection of blood samples at night entails determination of lower concentrations. It is unclear if the potential evening doses of administered cyclosporine and tacrolimus should be higher to maintain the optimum immunosuppressive effect [90]. Another clinical study evaluated the concentration of cyclosporine in the patients’ blood after liver transplant administered 140–150 mg of the drug in the form of an hourly intravenous infusion in the morning or at night. It was proven that cyclosporine concentration in the 8th hour after drug administration (identified as “C trough”) was lower in the case of drug administration at night. It was accompanied by the increase in the nighttime clearance of cyclosporin compared with the clearance during the day [91]. The trend of reaching higher immunosuppressant concentrations in the case of administration in the morning hours was also demonstrated in another clinical study carried out on 20 patients—liver recipients, during the first two weeks after the transplant. The patients were given cyclosporine microemulsion administered orally every 12 h. Blood samples were collected 2 h after administration of the morning and evening doses. Cyclosporine concentration in the 2nd hour after drug administration was significantly higher after the morning dose than after the evening dose, while the “Cthrough” concentrations and AUC values did not differ significantly for a.m./p.m. drug administration [92]. As mentioned above, aminoglycosides are also drugs subjected to TDM. Numerous experimental studies demonstrated that the nephrotoxicity of aminoglycosides, evaluated by the excretion of renal enzymes (*N*-acetyl-β-D-glucosaminidase and β-galactosidase), cortical and tubular cell lesions, blood urea nitrogen and serum creatinine level, and creatinine clearance, was maximal when the aminoglycoside was injected in the resting (day) compared with activity (night) phase in rats. Similar daily differences in aminoglycoside toxicity have been found in human patients. Renal toxicity was more frequently demonstrated when gentamicin or tobramycin were injected once daily during the night (rest) periods. Aminoglycosides are excreted mainly by the kidney, and accumulation of these antibiotics in the renal tubular cells is the main factor contributing to their nephrotoxicity. Chronobiological variations in glomerular filtration and other renal functions related to the saturable mechanisms responsible for transfer aminoglycosides into the proximal renal tubular epithelial cells may account for the lower drug elimination during the rest phase, producing higher renal accumulation and toxicity. These phenomena also underlie potential diurnal differences in the determination of blood aminoglycoside concentrations [93,94].

To summarize, there are documented reports regarding chronopharmacokinetic differences in multiple drugs covered by the TDM procedure, which emphasizes greatly the need to collect samples for the evaluation of drug concentration taking into consideration the potential differences resulting from the chronopharmacokinetic aspects of these drugs. It seems that the official TDM recommendations and protocols should, therefore, provide for the differences in drug concentrations conditional upon chronopharmacokinetic phenomena and take them into account when interpreting the obtained result.

## Figures and Tables

**Table 1:** The examples of pharmacotherapy in line with chronopharmacological recommendations.

Area	Disease	Chronobiological Aspects in the Pathophysiological Description	Chronopharmalogical Recommendations
Cardiovascular system	Hypercholesterolemia	Hepatic cholesterol synthesis is intensified in the evening and during the night	Administration of statins in the evening [1,2]
	Acute cardiovascular episodes (acute coronary syndromes and stroke)	There is an increased risk of acute cardiovascular events in early morning hours between 6.00 a.m.–noon.	The drugs should be administrated in the morning hours, e.g., beta-adrenoreceptor antagonists, since the morning dosing of the drugs is correlated with the morning peak of sympathetic activity [1,2,12,16]
	Hypertension	Among patients with primary arterial hypertension, the population of “dippers” (patients showing a decrease in nighttime RR value) or “nondippers” (patients with no expected night decrease in RR value), as well as patients characterized by the phenomenon of “morning surge” (an excessive morning surge in the value of RR) can be distinguished	“dippers”—antihypertensive drug administered in the morning
			“nondippers”—antihypertensive drug given in the evening or 2/3 of the dose in the evening and 1/3 in the morning
			“dippers” + “morning surge”—antihypertensive drug in the morning or 1/2 dose in the morning and 1/2 in the evening
			“nondippers” + “morning surge”—antihypertensive drug given in the evening or 2/3 of the dose in the evening and 1/3 in the morning [1,2,15,16]
Respiratory system	Bronchial asthma	There is an increased risk of an asthma attack between 4.00 a.m. and 6.00 a.m. It is usually correlated with allergic nasal congestion and sneezing, which tends to be greatest during night hours	The administration of an asthma medication in the early morning [1,2]
Digestive system	Peptic ulcer disease	There is an increase in gastric secretion in the evening and at night	H2 antihistaminics should be administrated at bedtime [1,2]
Nervous system	Epilepsy	There is an increased risk of a seizure between 6.00 and 7.00 a.m.	Antiepileptic drug should be used in the early morning hours, before getting out of bed [1,2]
	Depression	The occurrence of seasonal—autumn and winter—depression is observed due to the lower insolation	Administration of antidepressant drugs (e.g., agomelatine, St. John’s wort preparations) may be beneficial in the autumn and winter period as a supplement to phototherapy [1,2]
	Insomnia	The sleep phases change with a certain phasing	Administration of hypnotic drugs in the form of therapeutic systems, pulsating the release of the active substance, which would ensure the continuity of sleep [1,2]
		Some type of insomnia is related to disturbances in the biological clock (“jet lag”; “shift work”)	Administration of melatonin or agomelatine as compounds that synchronize the biological clock via MT1 and MT2 receptors may have beneficial effects [1,2]
	Pain	Pain sensations, especially of a neuropathic nature, increase between 3.00 a.m. and 8 a.m.	Administration of an additional dose of analgesic at bedtime or administration of a higher dose in the evening [1,2]
	Migraine	Migraine episodes often occur between 8.00 a.m. and 10 a.m. and are preceded by prodromal disturbances in the early morning hours	Administration of triptans in the early morning hours [1,2]
Endocrine system	Addison’s disease	Addison’s disease is an autoimmune condition resulting in complete deficiency of aderenal steroids and requiring replacement therapy with gluco- and mineralo-corticoids	The substitutive cortisol therapy is based on the administration of a high dose in the morning and low dose in the afternoon, which is to reflect the circadian variability of the hypothalamic-pituitary–adrenal axis [32]
	Diabetes	Blood levels of both insulin and counterregulatory hormones (growth hormone, cortisol) change in a circadian rhythm. In the middle of the night, there is a peak secretion of growth hormone, followed by a surge in cortisol, and these hormonal changes contribute to hyperglycemia. In diabetic patients, due to the lack of insulin action, the “dawn phenomenon”—morning hyperglycaemia—occurs between 4 a.m. and 8 a.m.	Conducting intensive insulin therapy, which normalizes the round-the-clock glycemic profile [33]
Locomotor system	Rheumatic arthritis Osteoarthritis	The symptoms intensify after waking up	Administration of an anti-inflammatory drug (NSAID) and cortisol in the morning before starting the daily activity of the patient [1,2]
Immune system	Infections	The susceptibility to a variety of pathogens (e.g., *Streptococcus pneumoniae, Listeria monocytogenes*, *Herpes,* and *Influenza* viruses) is higher at the beginning of the resting phase and many immunorelated processes show diurnal variations.	The future direction of antimicrobial and anti-inflammatory therapy is time-of-day-specific administration of pharmacologic agents aimed at modulating the immune response [34]
Cancer		The phenomenon of cancer chronobiology is rapidly explored. The diurnal variability of promotion and progression of carcinogenesis at the molecular level (e.g., DNA synthesis and cell proliferation, angiogenesis, and blood flow through the tumor) has been demonstrated for various tumors.	Experimental and clinical studies increasingly show positive associations between the circadian clock and drug response in cancer patients. The aim of cancer chronopharmacotherapy is to improve the efficacy of drugs and to minimize adverse effects by administering chemotherapeutic drugs at the appropriate time of day [31]

**Table 2:** The examples of drugs subjected to the TDM procedure [31,55,58,59,60,61,62,63,64,65,66,67].

Class of the Drugs	Examples
Antibiotics	aminoglycosides: gentamicin, amikacin, tobramycin
	glycopeptides: vancomycin
Antifungal drugs	triazoles: itraconazole, voriconazole, posaconazole, 5-fluorocytosine
Antiviral drugs	antiretroviral drugs in HIV-infected patients (protease inhibitors and the non-nucleoside reverse transcriptase inhibitors)
Neurological and psychiatric drugs	antiepileptic drugs: phenytoin, phenobarbital, carbamazepine, valproate tricyclic antidepressants (and its metabolites): imipramine (and desipramine), amitriptyline (and nortriptyline), clomipramine (and *N*-desmethyl-clomipramine) mood stabilizers: lithium, carbamazepine, valproate antipsychotics: amisulpride, clozapine, olanzapine, fluphenazine, haloperidol, perazine, perphenazine, thioridazine selective serotonin reuptake inhibitors: citalopram
Cardiovascular drugs	digoxin
	antiarrhythmic drugs: procainamide, amiodarone, flecainide,
Antiasthmatic drugs	theophylline,
Immunosuppressants	cyclosporin, sirolimus, tacrolimus,
Anticancer drugs	methotrexate, 5-fluorouracil, paclitaxel, docetaxel, imatinib
